# Functional Starch-Based Biopolymer Coatings for Sustainable Packaging Applications

**DOI:** 10.3390/polym17243303

**Published:** 2025-12-13

**Authors:** Teodora Lukavski, Josip Bota, Ivan Budimir, Katarina Itrić Ivanda, Rahela Kulčar, Marina Vukoje Bezjak

**Affiliations:** University of Zagreb Faculty of Graphic Arts, Getaldićeva 2, 10000 Zagreb, Croatia; teodora.lukavski@grf.unizg.hr (T.L.); katarina.itric.ivanda@grf.unizg.hr (K.I.I.); rahela.kulcar@grf.unizg.hr (R.K.); marina.vukoje@grf.unizg.hr (M.V.B.)

**Keywords:** coating, sustainability, biopolymer, natural plasticizers, thermochromic printing ink

## Abstract

Polymer waste poses significant environmental challenges and polymers’ replacement with biodegradable biopolymers is therefore crucial. Protective coatings are recognized as a problem in the graphics industry, since they are based on synthetic polymers that are not biodegradable. To be effective, coatings in this industry must meet several key criteria, including stability, functionality, recyclability, and a suitable shelf life. One potential alternative is starch, a highly abundant biopolymer that has been researched for its application in coating preparation. However, its poor mechanical properties have limited its use in this area. This paper investigates the functional properties of starch-based biopolymer coatings enhanced with D-sorbitol and bioactive components: trans-ferulic and L-ascorbic acid. The impact of these additions on the mechanical stability and UV protection potential of the coatings was evaluated. Thermochromic pigments are highly sensitive to UV radiation, making them a useful indicator for evaluating the UV protection factor of a given coating. The colour change of the coated thermochromic prints after UV irradiation was quantitatively evaluated using spectrophotometric analysis. Mechanical properties were assessed through tensile strength, elongation at break, burst strength, and folding endurance. Overall, coatings increased burst strength and improved key mechanical properties depending on the additive combination.

## 1. Introduction

Synthetic polymer waste poses a big environmental challenge due to its non-biodegradable and voluminous nature, high transportation costs, and energy used for the recycling process, as well as problems that may arise during the recycling process [1]. Such waste, when recycled, becomes a low-value material with lower quality and functionality traits. Notably, over 90% of polymer materials come from new raw materials originally from non-renewable sources. This unsustainable trend is expected to worsen, with production projected to double in the next 20 years and quadruple by 2050 [2]. In light of these concerns, it is necessary to develop alternative material solutions that minimize the use of non-renewable materials and promote the recycling and reuse of waste polymers [3]. Increasingly, industries are embracing the principles of the circular economy, where waste is reimagined as a valuable raw material, serving as the starting point in a closed-loop process [4]. This approach not only reduces dependence on non-renewable and natural resources but also fosters the development of more environmentally friendly products. A notable example is agro-industrial waste, which has been identified as a valuable, bioactive compound with film-forming properties [5]. However, if left untreated and unutilized, agro-industrial waste could also pose an environmental risk. Conversely, when economically exploited as an initial input material in a process, it can provide a range of benefits [6,7]. Additionally, biopolymer coatings offer the advantage of being lower-cost. However, their characteristics can vary significantly depending on the industry in which they are applied. In the graphics industry, it is essential to identify the optimal biopolymer coating. The majority of research on biopolymer materials originates from the food and medicine sectors. The food industry, in particular, faces a pressing challenge: synthetic polymer packaging poses a significant risk to consumers, necessitating the development of safer alternatives. To achieve sustainable packaging solutions, it is crucial to focus on materials that require minimal virgin resources and can be recycled or reused from readily available materials [8]. Biopolymers in the food industry are utilized as edible films, coatings, and packaging, while the medical industry uses them as a material for developing biocompatible implants, wound dressings, tissue engineering scaffolds, drug delivery systems, and healing products [9]. Since 2000, the number of research papers about natural biopolymers has substantially increased, gaining interest from researchers all around the world [10]. Protective coatings in the graphics industry are mainly based on synthetic polymers, which pose a significant challenge. While progress has been made on the transition from solvent-based coatings to water-based alternatives, further research and development are needed to completely replace synthetic coatings with biopolymer-based ones [11]. This involves investigating the potential of natural polymers, such as proteins, starch, cellulose, and other polysaccharides, which constitute the biopolymer group [12]. Starch is one of the most promising natural polymers for the future due to its favourable price, abundance, and thermoplastic properties. It has great potential as a material for the production of edible films due to its film-forming properties. Such films made from starch are known for their higher mechanical strength and good barrier properties. Specifically, films made from tapioca starch are notable for their non-toxic, odourless, tasteless, colourless, and biodegradable characteristics [13].

This research focuses on starch-based biopolymers, particularly those derived from tapioca starch, due to their exceptional biocompatibility and biodegradability, making them an attractive green substitute for non-biodegradable films. By integrating biodegradable polymers into our products, we can mitigate the environmental impact of non-biodegradable films [14]. Despite the numerous advantages of biopolymers, including sustainability, biodegradability, and recyclability, they still present significant drawbacks due to their poor mechanical and barrier properties. To overcome these limitations, biopolymers can be modified by the incorporation of certain compounds. The mechanical properties, gas barrier, and flexibility of packaging materials can all be enhanced through the addition of additives [15]. For example, plasticizers, such as sorbitol [16], glycerol [17], and polyethylene glycol, alter the mechanical characteristics of hydrophilic films, thereby imparting greater flexibility to films and coatings [18,19]. This prevents the film from chipping or breaking when handled and stored later. Glycerol, as a plasticizer, enhances flexibility, but it tends to lower the thermal stability of the films [20]. In contrast, sorbitol-plasticized films demonstrate higher tensile strength and thermal stability, but the lowest water vapour permeability and lowest film solubility [21], all of which may contribute to improved stability of films containing active ingredients—particularly due to their reduced permeability and enhanced thermal resistance.

Thermochromic (TC) inks are sensitive and known for their susceptibility to UV degradation and heat [22], and it is crucial that graphic products printed with TC inks are stable during their life cycle. UV stabilizers used in packaging applications are often a mixture of hazardous chemical substances [23,24]. Thus, it is of great importance to develop a toxic-free biodegradable coating that provides good UV protection. Adding active compounds, such as antioxidants or other active ingredients, can significantly enhance the properties of the coating [25,26,27]. Coatings used in the graphics industry must fulfil many requirements, such as functionality, stability, and having a certain shelf life. Beyond the standard plasticizers discussed previously, certain organic acids also exhibit plasticizing properties. Phenolic acids can change the properties of starch by chemical reaction [28]. In addition to its great antioxidant properties, ferulic acid has been found to significantly enhance the mechanical properties of films, such as elongation at break, elastic modulus, and tensile strength [29,30]. Moreover, its derivatives have recently been shown to possess plasticizing properties, effectively improving the flexibility of polylactic acid (PLA) and polycaprolactone (PCL) [31]. Ascorbic acid, another compound with notable plasticizing capabilities, has been used as a viable alternative to traditional plasticizers, resulting in enhanced mechanical, thermal, and optical properties in cured films [32,33].

The aim of this research is to investigate the functional properties of starch-based, biopolymer coatings applied to a fibre-based substrate, which is derived from grape agro-industrial waste, and intended for use in sustainable packaging applications. D-sorbitol was used as a plasticizing agent, while the influence of ferulic and ascorbic acid as alternative plasticizers was also evaluated. Additionally, the UV stability and mechanical properties of the biopolymer coatings were assessed. The unique sensitivity of thermochromic pigments to UV radiation makes them an ideal indicator for monitoring the effects of UV exposure on the biopolymer coatings, providing valuable insights into their durability and potential for sustainable packaging applications. The novelty of this research lies in the development and evaluation of novel starch-based biopolymer coatings, modified with D-sorbitol, ferulic acid, and ascorbic acid, to create UV-protective layers for thermochromic prints and enhance the mechanical properties of sustainable packaging materials. This innovative approach, integrating biopolymer materials into graphics technology and packaging applications, offers a promising solution for a more environmentally friendly and eco-conscious industry

## 2. Materials and Methods

### 2.1. Materials

For the evaluation of the UV-protective aspects of biobased coatings, as a printing substrate for the production of thermochromic prints, a pressure-sensitive label (PSL) was used. The composition of the label includes facestock consisting of 15% grape waste from wine-making processes, 40% post-consumer recycled fibres, and 45% virgin wood pulp, ensuring high-quality natural paper. The facestock is made from FSC^®^ certified paper, with a basis weight of 90 g/m^2^, in accordance with ISO 536 [34]. The adhesive used in PSL is rubber-based, while the liner is also made from FSC^®^ certified paper, with a basis weight of 69 g/m^2^, in accordance with ISO 536 [34,35]. The used thermochromic ink is a reversible leuco dye-based ink. The TC ink is a commercially available screen-printing ink and has an activation temperature (T_A_) of 28 °C. Before reaching the T_A_, the thermochromic print is coloured in green. When the print reaches the T_A_, it changes its coloration to a neon yellow. The type of ink with the mentioned properties is also called body heat-activated ink because it changes colour when exposed to human body temperature. Thermochromic inks with this activation temperature are often used in security printing, interactive packaging, temperature indicators on drinkware, and novelty items. Printing on PSL samples was carried out using a semi-automatic screen-printing device (Holzschurer KG, Wuppertal, Germany), under laboratory conditions. The prints were produced in full tone, using a mesh with a density of 62 threads/cm.

For the evaluation of the mechanical properties of the produced coating, an uncoated, matte smooth printing paper with a basis weight of 90 g/m^2^ was used. The used paper was made of 15% grape waste from wine-making processes, 40% post-consumer recycled fibres, and 45% virgin wood pulp.

#### 2.1.1. Coating Preparation for Assessing the UV Protection Factor

Coatings were prepared by weighing out tapioca starch (T) (GreenLab, Zagreb, Croatia) and D-sorbitol (S) (CARLO ERBA Reagents, Milan, Italy) as presented in Table 1. Distilled water was added until the total volume reached 100 mL. It was then transferred to a magnetic stirrer with a heater (IKA RCT basic IKAMAG^®^ safety control, Staufen, Germany), where it was heated until the temperature reached 80 °C, i.e., until the gelatinization of tapioca starch occurred. After the gelatinization, it was cooled down to room temperature. Coatings containing trans-ferulic acid (F) (Sigma-Aldrich, Merck, St. Louis, MO, USA) and L-ascorbic acid (A) (Sigma-Aldrich, Merck, Germany) were prepared in the same way, with bioactive components added to tapioca starch in various percentages (Table 1) after gelatinization and cooling to room temperature.

In coatings containing acids, the pH value had to be adjusted after the addition of acids, using Na_2_HPO_4_ (Fischer Scientific, Pittsburgh, PA, USA) and NaH_2_PO_4_ (Gram-Mol d.o.o., Zagreb, Croatia). As a weak base, Na_2_HPO_4_ was used to control the rise in the pH value to a basic range. On the contrary, NaH_2_PO_4_, as a weak acid, was used for controlled lowering of the pH value. The goal was to achieve a coating with a pH of 6, which is in a slightly acidic, close-to-neutral range and is preferred for coatings due to achieving greater stability of the starch matrix, which results in more favourable viscosity. Prepared coatings were used for coating of thermochromic prints in order to test their UV potential. Thermochromic prints were coated using a coater (RK K202 control coater, Litlington, UK), employing a wire-wound bar with a 0.30 mm wire thread, producing a layer of 24 µm thick film.

#### 2.1.2. Coating Preparation for Assessing Its Influence on the Mechanical Properties of Fibre-Based Substrate

In addition to the coatings utilized in previous UV stability tests, additional coatings (Table 2) were formulated after the UV-protective testing on thermochromic prints was completed. In order to better observe the impact of the coatings on the mechanical properties, i.e., which component of the coating exhibits higher mechanical properties, the coatings that demonstrated better performance in earlier experiments were separated into their individual components. Coatings were prepared using the same procedure described in Section 2.1.1. Prepared coatings were used for the coating of unprinted paper. Papers were coated using a coater (RK K202 control coater, Litlington, UK), employing a wire-wound bar with a 0.30 mm wire thread, producing a layer of 24 µm thick film.

All tested samples after application on paper and drying were conditioned for 24 h at the required temperature (23 °C ± 1 °C) and relative humidity (50% RH ± 2%) in accordance with the ISO 187:2022 standard [36].

### 2.2. Methods

#### 2.2.1. Accelerated Ageing Tests

For the evaluation of the UV-protective properties of the coatings, an uncoated TC print and coated thermochromic prints on PSLs were exposed to UV irradiation in a Solarbox 1500e device (CO.FO.ME.GRA, Milan, Italy), which simulates conditions of the environment in an open or closed space, with controlled temperature and radiation. The filtered xenon light used in the device provides radiation predominantly within the UV-A spectral range (315–400 nm), while visible and near-infrared radiation are also present. The samples were irradiated for 5 and 8 h at a temperature of 50 °C and a total irradiance of 550 W/m^2^.

#### 2.2.2. UV-Vis Spectroscopy

In order to evaluate the colour change of coated thermochromic prints after exposure to UV irradiation, an Ocean Optics USB2000+ spectrometer r (Ocean Optics, Orlando, FL, USA) with a 30 mm wide integrating sphere was used. Measurements were performed under (8:di) geometry (diffuse illumination, specular component included). The OceanView 2.0 software (Ocean Optics, Orlando, FL, USA) was used to calculate the CIELAB colour values from the recorded spectra. The samples were subjected to heating and cooling on a full-cover water block (EK Water Blocks, EKWB d.o.o., Komenda, Slovenia). Measurements were taken at 1 nm intervals across the spectral range of 370–730 nm. The total colour difference between coated printed PSL samples at 20 °C and 40 °C was determined using the CIEDE2000 formula [37].

A Shimadzu UV-1900 UV–Vis double-beam spectrophotometer (Kyoto, Japan) with a spectral bandwidth of 1 nm was employed to record the absorbance spectra of the prepared coatings in the UV range (190–400 nm). The instrument utilized a deuterium lamp as a light source.

#### 2.2.3. Tensile Strength and Elongation at Break

The determination of tensile strength and elongation at break was conducted using a Karl Frank GmbH tensile testing apparatus in compliance with the ISO 1924-2:2008 standard [38]. Test samples were prepared with dimensions of 250 × 15 mm, clamp separation was 180 mm, and a testing speed of 20 mm/min. The breaking force (N/mm) at the point of sample failure and the corresponding elongation (%) were recorded. For each sample, tensile properties were evaluated with 10 samples in both fibre orientation directions: the machine direction (MD) and the cross direction (CD).

#### 2.2.4. Bursting Strength

The bursting strength of the samples was determined using a Lorentzen & Wettre Bursting Strength Tester in accordance with ISO 2758:2014 standard [39]. This method evaluates the resistance of paper to rupture under increasing hydraulic pressure applied through a flexible diaphragm. Circular test samples with a diameter of 100 mm were clamped so that the effective test area corresponded to a Ø 50 mm diameter, with an elastic diaphragm of Ø 33.1 mm in diameter. During testing, pressure gradually increased until rupture occurred, and the maximum pressure at the point of failure was recorded as the bursting strength. Each sample was tested by repeating the test 10 times.

#### 2.2.5. Folding Endurance

The folding endurance of the samples was measured using the Karl Frank GmbH Folding Tester for paper and cardboard in accordance with the procedure specified in ISO 5626:1993 standard [40]. Samples were cut to dimensions of 140 × 15 mm. During the test, the plate was moved alternately back and forth, subjecting the sample to repeated double folding under a constant tensile force of 9.81 N. Folding continued until the sample ruptured, at which point the number of double folds to failure was automatically recorded by the device. For each sample, ten measurements were performed in the machine direction (MD), and the results were expressed as the mean number of double folds.

#### 2.2.6. Statistical Analysis

For the evaluation of colour differences, CIEDE2000 values were calculated from the measured CIEL*a*b* values before and after UV radiation (5 h and 8 h). For each formulation, three independent measurements were performed. Due to the small sample size (n = 3), the nonparametric Kruskal–Wallis test was performed to determine whether the type of coating had a significant influence on CIEDE2000 (*p* < 0.05).

The experimental results of mechanical properties were statistically analyzed using the software package Statistica 13 (StatSoft, Tulsa, OK, USA). For all statistical tests performed (Kolmogorov–Smirnov test, Kruskal–Wallis test, and Dunn’s post hoc test with Bonferroni correction), a significance level of *p* < 0.05 was adopted, as this is standard practice in studies of this type.

## 3. Results and Discussion

### 3.1. Spectroscopic Assessment of UV Exposure Effects and Protective Behaviour of Coatings

**Figure 1:**
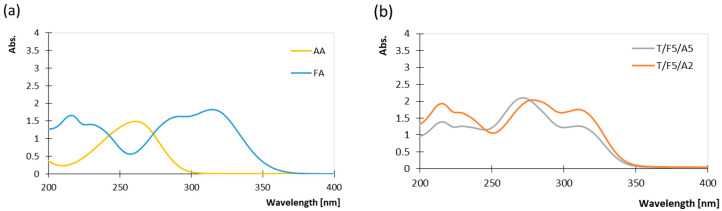
(**a**) Absorption spectra of ascorbic acid in water at pH 3.2 (AA) and ferulic acid in water at pH 3.8 (FA). (**b**) Absorption spectra of tapioca starch-based coating containing ferulic acid and different ratios of ascorbic acid at pH 6.

The absorption spectra of ascorbic and ferulic acid in aqueous solutions are presented in Figure 1. The absorption spectrum of ascorbic acid shows a strong absorption band around 270 nm [41]. Ferulic acid [42] exhibits absorption maxima around 215, 250–280, and around 315 nm.

The absorbance spectra of the tapioca starch-based coating containing ferulic acid and ascorbic acid exhibit three characteristic absorption regions: a band at approximately 215 nm attributed to ferulic acid, a band at 240–290 nm attributed to the combined contributions of ascorbic acid and ferulic acid (i.e., overlapping of absorption bands), and a second, broader band between 300 and 320 nm primarily associated with the cinnamic chromophore of ferulic acid (Figure 1b). Increasing the proportion of ascorbic acid in the coating (T/F5/A5) causes a change in the absorption spectra, namely, it is visible that the absorption peak of ferulic acid at 315 nm is masked, and that the peak between 250 and 290 nm is sharpened. In addition, the pH of the solution significantly influences the absorption spectrum of ascorbic acid, causing a shift in its maximum absorption wavelength. As the pH increases from acidic to alkaline, its maximum absorption wavelength typically moves from lower to higher wavelengths. This behaviour can be observed in the prepared tapioca starch-based coating, in which there is a shift in the absorption peak from 260 nm in water solution (pH 3.2) towards 275 nm (coating of pH 6) [43]. The opposite behaviour is observed in the case of ferulic acid, where the absorption range is transferred to lower wavelengths in coatings with higher pH values (Figure 1a,b).

**Figure 2:**
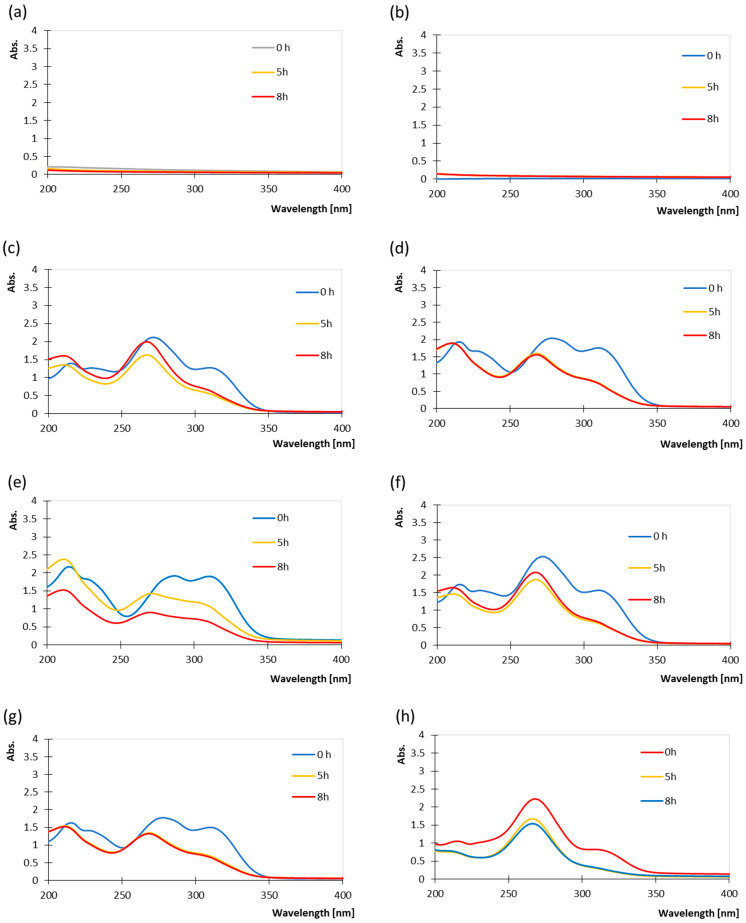
Absorption spectra of coatings (**a**) T; (**b**) T/S; (**c**) T/F5/A5; (**d**) T/F5/A2; (**e**) T/F5/S; (**f**) T/F5/A5/S; (**g**) T/F5/A2/S; (**h**) T/F2/A5/S (all measured at pH 6) monitored over 5 and 8 h of UV exposure.

The absorption spectra of the prepared coatings in the UV range are presented in Figure 2a–f. The absorption spectra of the tapioca-based coating do not show any absorbance across the scanned wavelength range (200–400 nm), since it lacks conjugated chromophores capable of strong UV absorption (Figure 2a). Upon UV irradiation the spectra remain essentially unchanged, with only negligible variations in the baseline absorbance.

The absorption spectra of the tapioca–sorbitol coating remain essentially featureless across 200–400 nm, also confirming the absence of strong conjugated chromophores in the matrix (Figure 2a). The tapioca–sorbitol coating also remains unchanged in the examined wavelength range after exposure to UV irradiation, which can lead to the conclusion that sorbitol mainly alters physical properties (plasticization, hygroscopicity) rather than introducing new photochemical pathways. According to Zahiruddin et al. (2019), tapioca starch films plasticized with sorbitol show good thermal resistance when compared to glycerol because the high molecular weight of sorbitol within the starch polymer chain creates a stronger film by reducing the molecular mobility of the polymer chains, thereby enhancing thermal stability [44]. The resulting film could aid in reducing microcracks from UV/heat stress, maintaining better encapsulation of the added ferulic and ascorbic acid, unlike more flexible glycerol films. In the case of the coating formulation with a lower ratio of ferulic acid (T/F2/A5/S, Figure 2h), it can be seen that the absorption spectrum is similar to that of ascorbic acid. The absorbance spectra of the coating containing ferulic acid and ascorbic acid were monitored over 5 and 8 h of UV exposure to evaluate the photodegradation behaviour of the coating system (Figure 2d–f). After prolonged exposure to UV irradiation (8 h), a pronounced decrease in absorbance was detected across the entire spectral range, indicating substantial degradation of both active components. The marked reduction in the 300–320 nm band suggests the breakdown of the ferulic acid conjugated system, while the diminished intensity near 270 nm reflects the oxidation and subsequent decomposition of ascorbic acid. Overall, extended UV irradiation leads to significant depletion of both ferulic and ascorbic acid within the coating.

The photodegradation of pigments in printed matter is primarily caused by photo-oxidation, which is induced by the absorption of ultraviolet (UV) radiation. The absorbed energy excites the dye molecules, leading to the formation of free radicals and reactive oxygen species. These radicals initiate a chain reaction of oxidation that irreversibly destroys the chromophoric groups responsible for colour, which manifests as a loss of intensity and a shift in the print’s hue. Antioxidants act as stabilizers by scavenging and neutralizing these free radicals. They interrupt the destructive oxidative chain reaction by donating hydrogen atoms, thereby stabilizing the radicals and preventing oxidative damage to the chromophores. Effective photostability is often achieved through synergistic systems, where antioxidants complement the action of UV absorbers, thus ensuring both the prevention of UV damage and the quenching of resulting radicals. Ferulic acid and ascorbic acid show significant potential as stabilizers owing to their established antioxidant capacity. Ferulic acid, as a potent phenolic radical scavenger, is promising in the quenching of peroxyl radicals and offers complementary UV absorption. Ascorbic acid provides potential in suppressing reactive oxygen species and neutralizing trace metal ions that serve as degradation photocatalysts. The results of this study, supported by other research, also indicate that the synergistic potential of these two acids, where ferulic acid can stabilize the ascorbic acid, offers a strong basis for the chemical protection of chromophores [45]. Concurrently, D-sorbitol represents a potential functional additive that contributes to system stability through physical mechanisms. Its hygroscopic properties make it a valuable humectant for maintaining the stability of the coating matrix, which indirectly slows down oxidation.

**Figure 3:**
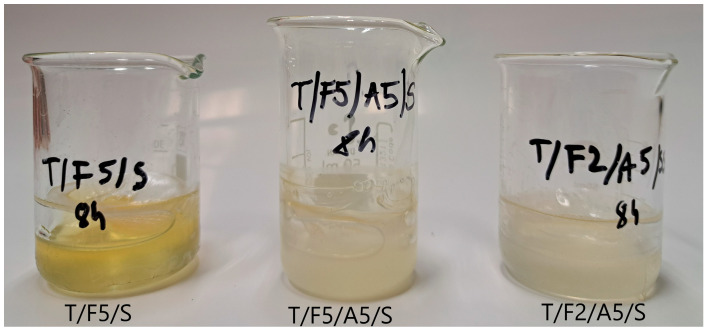
Visual appearance of coatings after exposure to UV irradiance for 8 h.

In this study, the samples were exposed to UV irradiation in the 315–400 nm range, corresponding mostly to UV-A. The coating applied to the samples contains ascorbic acid, which absorbs strongly around 270 nm and therefore does not directly absorb the UV-A wavelengths emitted by the chamber. However, ascorbic acid contributes to protection in another way: it acts as a powerful antioxidant that neutralizes reactive oxygen species generated by UV exposure, thereby slowing oxidation-driven degradation of the material. It also helps stabilize ferulic acid, preserving its photoprotective activity for longer, resulting in colour stability as well (Figure 3). As seen from Figure 3, during 8 h of exposure to UV irradiance, the coating that does not contain ascorbic acid (T/F5/S) develops a yellow coloration, in contrast to the coatings containing ascorbic acid. Since all coatings presented in Figure 3 contain sorbitol, it can be seen that it does not affect the UV stability of the coatings.

**Figure 4:**
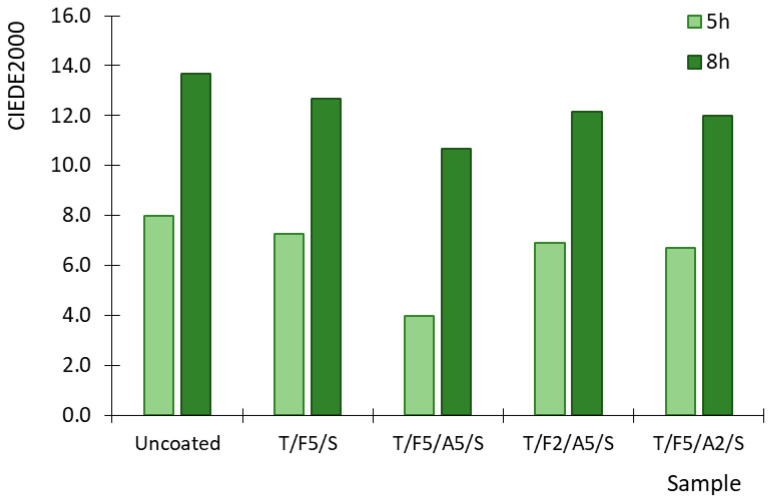
Colour difference (CIEDE2000) between uncoated and coated samples before and after 5 and 8 h exposure to UV radiation, measured at 20 °C.

Ferulic acid itself has absorption peaks at approximately 290 nm and 325 nm, with its absorption diminishing by 350 nm (Figure 1b), meaning it can partially attenuate the lower wavelength of UV-A spectrum. Consequently, the coating provides limited direct UV absorption—mainly around 315–325 nm—while wavelengths above 350 nm remain largely unfiltered; nevertheless, the antioxidant action of the used bioactive compounds enhances the overall resistance to UV-induced oxidative damage. These findings are supported by the colour determination of thermochromic prints after exposure to UV irradiance presented in Figure 4. Figure 4 shows the influence of UV irradiation on the colour difference for both the uncoated print and prints coated with the formulations listed in Table 1. The used thermochromic ink was selected for optical testing because it has a high colour density, and therefore, it is easier to visually assess the colour change after exposure to UV irradiation.

After 5 h of exposure to UV radiation, all samples showed a change in coloration. Moreover, 8 h of UV exposure produced significant changes in the print coloration. The lower value of CIEDE 2000 indicates lower colour change, with values below 3 indicating a small and negligible perception of colour difference [46]. The greatest change in colour was observed for uncoated samples in both cases of UV exposure (5 h and 8 h). 

Comparisons with coated samples reveal that certain formulations effectively reduce colour differences. The T/F5/A5/S coating provided the most pronounced protection. Notable improvements were also observed with the T/F5/A2/S and T/F2/A5/S coatings. In contrast, the T/F5/S coating showed minimal protection, displaying values comparable to those of the uncoated sample.

After 8 h of ageing, the uncoated sample showed the largest colour difference, confirming the extreme sensitivity and instability of TC prints when exposed to UV radiation. The coated samples exhibited lower CIEDE2000 values, with T/F5/A5/S providing the greatest protection, consistent with the results after 5 h of ageing. Higher difference values were found in T/F5/S, while the other coatings exhibited similar values. Although the protective effect of the coatings decreased with longer UV exposure, all coated samples still showed lower values than the uncoated sample. These results indicate that the formulation of the coatings used enhances the UV stability of TC prints, with certain formulations providing superior protection, offering potential as a protective measure, although their effectiveness decreases with prolonged exposure.

Although numerical differences in CIEDE2000 values between coatings appear small, statistical analysis confirmed a significant effect of coating formulation on colour stability. The Kruskal–Wallis test showed significant differences among coatings after 5 h (H (4) = 11.33; *p* = 0.023) and after 8 h of UV radiation (H (4) = 10.27; *p* = 0.036). The greatest colour difference was observed in the uncoated sample (ΔE ≈ 7.9 after 5 h and ≈13.6 after 8 h), while all coatings had lower ΔE values. The lowest values were recorded for T/F5/A5/S coating (≈4.0 after 5 h and ≈10.7 after 8 h). Despite the limited number of replicates, the results clearly indicate that coatings improve the UV stability of thermochromic prints.

In previous research by Sharma et al. (2020), it was shown that incorporation of ferulic acid into poly(lactide)–poly(butylene adipate-co-terephthalate) (PLA-PBAT) blends resulted in composite films exhibiting enhanced tensile strength, improved antibacterial activity, and superior UV-light barrier properties [29]. This confirms the multifunctional role of ferulic acid in enhancing both the mechanical performance and photostability of biopolymer materials. Ascorbic acid is an effective antioxidant that scavenges free radicals and inhibits oxidative degradation in polymers. However, its sensitivity to UV light and heat limits long-term stability. In UV-protective coatings, it can provide short-term antioxidative benefits, but stabilization strategies such as encapsulation or combination with other stabilizers like ferulic acid are needed to maintain effectiveness. Studies have shown that combining ferulic acid with vitamins C and E enhances UV protection and reduces oxidative damage [41].

The lowest colour change occurring at a ratio of 5% ferulic acid to 5% ascorbic acid indicates that this ratio provides the optimal synergistic effect in suppressing photo-oxidative degradation. When used together, ferulic and ascorbic acids act synergistically, where the total protective effect is greater than the sum of their individual effects.

### 3.2. Mechanical Properties

**Figure 5:**
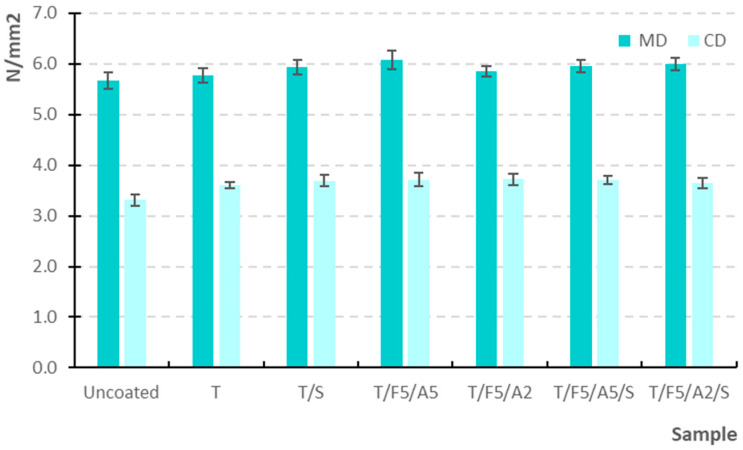
Tensile strength (MD and CD) of uncoated and coated samples on paper substrate.

The influence of the newly formulated coatings on the mechanical properties of the grape-based paper substrate was evaluated through tensile strength (Figure 5), elongation at break, bursting strength, and folding endurance in accordance with relevant ISO standards.

#### 3.2.1. Tensile Strength (MD and CD)

All coated samples exhibited higher tensile strength compared with the uncoated substrate in both the machine direction (MD) and the cross direction (CD) (Figure 5). In the MD, tensile strength increased from 5.66 N/mm^2^ for the uncoated paper to values between 5.78 and 6.1 N/mm^2^ for the coated samples, with the highest strength observed for the T/F5/A5 formulation. The CD values followed a similar trend, rising from 3.31 N/mm^2^ in the uncoated sample to 3.61 to 3.72 N/mm^2^ in coated papers.

These results show that the primary reinforcing effect originates from the tapioca starch matrix, which forms a continuous surface film that improves fibre bonding and reduces micro-defects [47,48]. Ferulic acid can form covalent (phenolic) crosslinked structures within starch-based or other biopolymer films, typically increasing tensile strength with only moderate effects on elongation, while also improving barrier properties. The literature reports increases in strength at optimal ferulic acid concentrations, although excessive levels may lead to phase separation and brittleness. This is consistent with our finding of a slight strength increase without a loss of ductility [49].

The higher tensile strength values in the MD than in the CD are expected due to the orientation of the fibres during papermaking; thus, the MD exhibits higher strength and stiffness, while the CD is more deformable [50,51].

#### 3.2.2. Elongation at Break (MD and CD)

**Figure 6:**
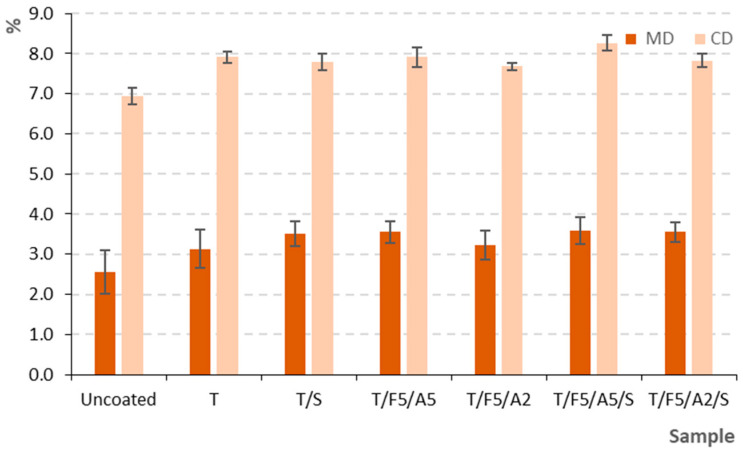
Elongation at break (MD and CD) of uncoated and coated samples on paper substrate.

The coated papers also demonstrated greater elongation at break than the uncoated samples (Figure 6). In the MD, elongation increased from 2.55% in the uncoated sample to 3.13–3.58% across the coated formulations, with the highest values recorded for T/F5/A5/S, T/F5/A5, and T/F5/A2/S. CD elongation exhibited a more pronounced increase, ranging from 6.94% in the uncoated sample to 7.68–8.27% in the coated samples, with the highest values again observed for T/F5/A5/S.

These findings indicate that ferulic and ascorbic acids contribute to greater ductility of the starch matrix, likely by modifying intermolecular interactions and enabling more extensive polymer chain mobility before rupture [30,49,52]. Sorbitol, as a known plasticizer, had the strongest influence on elongation, significantly enhancing flexibility, especially in the CD [53,54]. However, this increase in ductility must be viewed together with its effect on folding endurance [55].

#### 3.2.3. Bursting Strength

**Figure 7:**
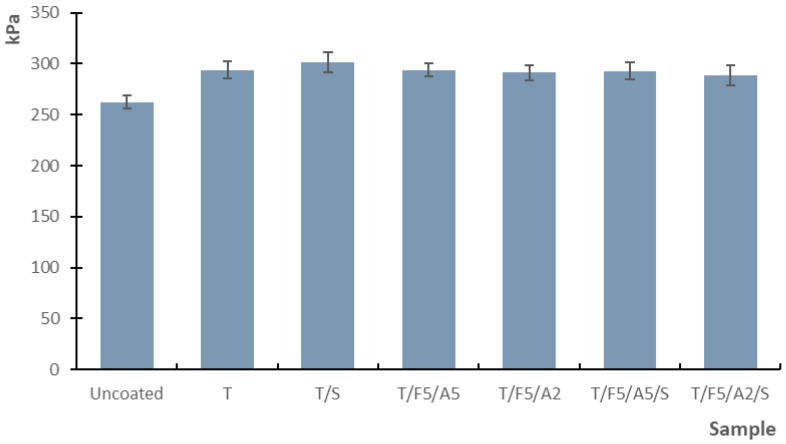
Burst strength of uncoated and coated samples on paper substrate.

Burst strength increased uniformly for all coated samples, from 262.1 kPa for the uncoated substrate to 288.4–301.4 kPa for the coated papers (Figure 7). The largest increase was observed with the basic tapioca coating (T), while formulations containing ferulic acid, ascorbic acid, and sorbitol showed slightly lower improvements.

The overall improvement can be attributed to the formation of a stronger and more cohesive film on the paper surface, which increases resistance to multidirectional pressure [56]. Because bursting strength depends primarily on structural reinforcement rather than chemical composition, the presence of the starch film itself is the dominant factor, explaining the small variation among formulations [43,57].

Burst testing loads paper biaxially and depends strongly on internal bonding and network density. Coating layers that enhance Z-direction cohesion often increase burst strength to a greater extent than they affect tensile strength. Partial penetration of the coating beneath the surface enlarges the fibre contact area and improves network cohesion. The literature reports correlations between burst strength, internal bonding, and coating penetration, with resulting increases in the Scott bond and burst values following surface sizing or coating with starch, either alone or in combination with other components. The increase in burst strength observed in our work following coating application is consistent with these findings [58].

#### 3.2.4. Folding Endurance

**Figure 8:**
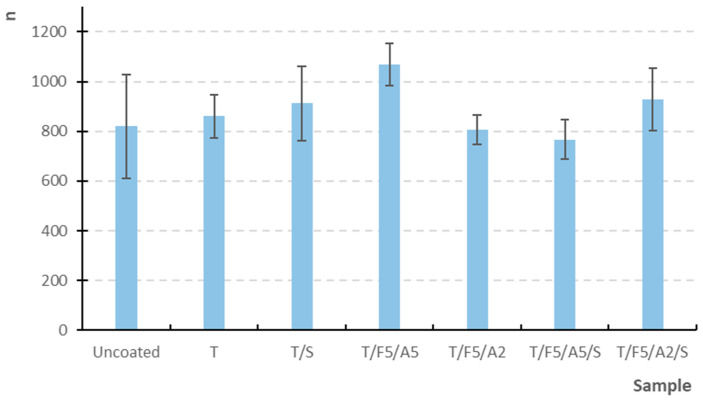
Double-fold endurance of uncoated and coated samples on paper substrate.

Folding endurance (Figure 8), however, showed the greatest sensitivity to coating composition. The uncoated paper withstood an average of 820 double folds, while coated samples exhibited values ranging from 760 to more than 1000 folds (Figure 8). The highest endurance was achieved by T/F5/A5, which averaged 1050 folds, indicating that the combined presence of ferulic and ascorbic acid significantly enhances folding endurance.

In contrast, the T/F5/A2 and T/F5/A5/S formulations demonstrated lower folding endurance despite their improved elongation, highlighting a well-documented phenomenon in plasticized films: increased elongation does not necessarily correlate with enhanced resistance to cyclic loading. Plasticization softens the film, enhancing its deformability under single-event loading, but hastening its failure under repeated bending [59,60].

#### 3.2.5. Statistical Analysis

In order to compare the mechanical properties of the uncoated substrate and coated papers, a statistical analysis was performed, comprising descriptive statistics of the arithmetic mean ($\bar{X}$) and the median (Med).

The descriptive statistical analysis (Table 3) showed that all paper properties, namely burst, tensile strength, and elongation at break, exhibited higher arithmetic mean and median values compared to the uncoated substrate, with the exception of folding endurance. The variances and standard deviations of the samples were also analysed, and their relatively low values indicate the precision of the measuring instruments used. The ranges between the minimum and maximum values across all samples are consistent, as none of the results deviate substantially from the arithmetic means.

Kolmogorov–Smirnov tests were performed on all samples, showing that all samples for all statistical parameters were normally distributed, with *p*-values greater than 0.20. To determine whether any pairs of samples were statistically significantly different, the applicability of the parametric one-way ANOVA test and the nonparametric Kruskal–Wallis test was examined. Although the Kolmogorov–Smirnov normality tests indicated that the data distributions did not deviate significantly from normality, the nonparametric Kruskal–Wallis tests were employed due to the small sample sizes of only n = 9 and n = 10 per group, as these tests are more robust than parametric methods under such conditions.

The results of the Kruskal–Wallis tests showed that, for all measurements of the mechanical parameters, there are pairs of samples whose medians were statistically significantly different (Table 4). In particular, the calculated *p*-values for bursting strength are *p* = 0.0001 < 0.05, while the *p*-values for all other tested mechanical properties are equivalent and amount to *p* = 0.0000 < 0.05. As a post hoc analysis, Dunn’s test with Bonferroni correction was performed to identify the specific sample pairs that were statistically significantly different (Table A1).

The post hoc Dunn–Bonferroni test for the bursting strength results identified statistically significant differences (*p* = 0.008737, 0.000023, 0.002469, 0.012231, 0.004702 < 0.05) between paper substrate and coated papers, except for T/F5/A2/S (Table A1) (*p* = 0.269667 > 0.05). The elevated variability observed in the T/F5/A2/S measurements may have concealed potential differences in mean values, as the inherently conservative Bonferroni correction decreases the statistical power to detect significant effects in datasets with high dispersion. No additional statistically significant differences were detected among the remaining sample pairs.

For the MD tensile strength, the uncoated paper substrate (having the lowest arithmetic mean value $\bar{X}\;=\;8.66$ and median value Med = 8.65, Table 3) was statistically significantly different from most other samples (samples T/S, T/F5/A5, T/F5/A5/S *p* = 0.037922, 0.000227, 0.030835 < 0.05 and T/F5/A2/S *p* = 0.001799 < 0.05), but not from samples T (*p* = 1.000000 > 0.05) and T/F5/A2 (*p* = 1.00000 > 0.05) (Table A1). Sample T/F5/A5, which exhibits the highest arithmetic mean value $\bar{X}$ = 9.29 and median value Med = 9.35 (Table 3), differs significantly from sample T (*p* = 0.010847 < 0.05), while no statistically significant differences were found between T/F5/A5 and the remaining samples (Table A1). Similar findings were obtained for the CD tensile strength perpendicular to the machine direction (Table A1). Uncoated paper substrate differs significantly from samples T/S, T/F5/A5, T/F5/A2, T/F5/A5/S, and T/F5/A2/S (*p* = 0.001215, 0.000479, 0.000143, 0.000195, 0.26983 < 0.05), while no significant difference was found between the uncoated paper substrate and sample T (*p* = 0.226808 > 0.05). Statistical analysis confirmed the trends observed in tensile strength results.

For the MD elongation at break, better results were observed for samples T/S, T/F5/A5, T/F5/A5/S, and T/F5/A2/S, which do not statistically significantly differ from each other (*p* > 0.05) (Table A1). Samples 0 and T show poorer performance compared to the other samples and are not statistically significantly different from each other (*p* = 1.0000 > 0.05), but both were statistically significantly different from most remaining samples (*p* < 0.05) (Table A1). For the CD elongation perpendicular to the machine direction, sample T/F5/A5/S shows the highest elongation ($\bar{X}$ = 8.27, Med = 8.30, Table 3) and statistically significantly differs from the uncoated paper substrate and T/F5/A2 (*p* = 0.000003, 0.037221 < 0.05 (Table A1). Sample T/F5/A5/S containing sorbitol and organic acids in the same ratios shows the best performance, indicating greater ductility.

Sample T/F5/A5 exhibits the best folding endurance in the machine direction, ($\bar{X}=$ 1068.60, Med = 1053.50) whereas sample T/F5/A5/S shows the lowest performance ($\bar{X}$ = 766.30, Med = 747.50, Table 3), and these two samples differ significantly (*p* = 0.000070 < 0.05) (Table A1), confirming the previously observed trend that the sorbitol-containing formulation demonstrates reduced resistance to cyclic loading.

## 4. Conclusions

The goal of this research was to investigate how bioactive compounds—trans-ferulic acid and L-ascorbic acid, enhanced with D-sorbitol, influence functional properties of starch-based biopolymer coatings. The research evaluated the effect of these formulated coatings on mechanical durability and assessed their ability to provide ultraviolet (UV) protection for thermochromic prints. The results of assessing the UV protection factor indicate that applying these coatings to TC prints enhances their UV stability, with the T/F5/A5/S formulation offering the most significant protection. A ratio of 5% ferulic and 5% ascorbic acid yields the optimal synergistic effect in suppressing photo-oxidative degradation. D-sorbitol contributed to system stability primarily through physical mechanisms, enhancing the stability of the polymer matrix. Mechanical properties were assessed by measuring tensile strength, elongation at break, bursting strength, and folding endurance. All experimental data were statistically analysed, and differences among various coatings were identified using Kruskal–Wallis tests. In both the machine and cross directions, all coated samples demonstrate higher tensile strength than the uncoated substrate, with the T/F5/A5 formulation exhibiting greater strength. This indicates that the tapioca starch matrix is the main contributor to the reinforcing effect, while additives such as ferulic acid, ascorbic acid, and sorbitol play a secondary role in the enhancement of tensile strength. Additionally, the coated samples also showed higher elongation at break compared to the uncoated samples, with the highest values obtained for T/F5/A5/S formulation. The elongation was most significantly influenced by sorbitol, a well-known plasticizer. All coated samples exhibit a consistent increase in burst strength, with the basic tapioca coating showing the greatest improvement. The highest folding endurance was observed in T/F5/A5-coated samples, suggesting that the presence of both ferulic and ascorbic acid enhances folding endurance. This may suggest that ascorbic acid, an alternative plasticizer known to improve flexibility in starch-based films, should be added at concentrations higher than 5% to achieve greater flexibility. Additionally, the ratio of the acids must be optimized to maximize the benefits of both. D-sorbitol is known to increase tensile strength but may slightly reduce flexibility when incorporated into coatings, a trend supported by the results showing a decreased resistance to cyclic loading—particularly in formulations containing sorbitol. This was especially evident in the T/F5/A5/S-coated samples, which demonstrated lower folding endurance.

Building on the promising outcomes of this research, future investigations will concentrate on developing novel biopolymer coatings that incorporate bioactive compounds in stable and optimized formulations. This will allow for the realization of coatings with superior UV stability and functional properties, poised to make a significant impact on the graphics industry’s pursuit of more sustainable solutions.

## Figures and Tables

**Table 1:** Coatings prepared for assessing the UV protection factor.

Coating Abbreviation	Tapioca Starch/g	Ferulic Acid/%	Ascorbic Acid/%	D-Sorbitol/%
T/F5/S	6	5	-	15
T/F5/A5/S	6	5	5	15
T/F2/A5/S	6	2	5	15
T/F5/A2/S	6	5	2	15

**Table 2:** Coatings prepared for assessing the mechanical properties.

Coating Abbreviation	Tapioca Starch/g	Ferulic Acid/%	Ascorbic Acid/%	D-Sorbitol/%
T	6	-	-	-
T/S	6	-	-	15
T/F5/A5	6	5	5	-
T/F5/A2	6	5	2	-
T/F5/A5/S	6	5	5	15
T/F5/A2/S	6	5	2	15

**Table 3:** Results of the descriptive statistical analysis for the mechanical properties.

Coating Abbreviation	Bursting Strength		Tensile Strength MD		Tensile Strength CD		Elongation at Break MD		Elongation at Break CD		Folding Endurance	
	$\bar{X}$	Med	$\bar{X}$	Med	$\bar{X}$	Med	$\bar{X}$	Med	$\bar{X}$	Med	$\bar{X}$	Med
Uncoated	262.44	262.00	8.66	8.65	5.07	5.05	2.55	2.50	6.94	7.10	819.90	759.00
T	292.78	293.00	8.82	8.80	5.52	5.50	3.13	3.10	7.91	8.00	860.40	872.00
T/S	300.56	302.00	9.08	9.10	5.65	5.65	3.50	3.50	7.79	8.00	912.80	931.00
T/F5/A5	294.56	296.00	9.29	9.35	5.68	5.60	3.55	3.55	7.91	8.00	1068.60	1053.50
T/F5/A2	292.67	293.00	8.95	8.90	5.69	5.70	3.22	3.20	7.68	7.80	805.50	793.50
T/F5/A5/S	294.00	294.00	9.10	9.10	5.68	5.70	3.58	3.60	8.27	8.30	766.30	747.50
T/F5/A2/S	288.44	285.00	9.17	9.20	5.58	5.55	3.55	3.60	7.83	7.90	928.50	894.50

**Table 4:** Results of the Kruskal–Wallis tests (N—sample size, H—statistic, df—degrees of freedom, *p*-value).

Mechanical Properties	N	H	df	*p*
Bursting strength	63	28.75	6	0.0001
Tensile strength MD	70	31.98	6	0.0000
Tensile strength CD	70	31.80	6	0.0000
Elongation at break MD	70	47.90	6	0.0000
Elongation at break CD	70	31.44	6	0.0000
Folding endurance	70	30.24	6	0.0000

## Data Availability

The original contributions presented in this study are included in the article. Further inquiries can be directed to the corresponding author.

## Nomenclature

A	L-ascorbic acid
CD	Cross direction
F	Trans-ferulic acid
MD	Machine direction
Na_2_HPO_4_	Sodium phosphate dibasic
NaH_2_PO_4_	Sodium phosphate monobasic
PCL	Polycaprolactone
PLA	Polylactic acid
PSL	Pressure-sensitive label
S	D-sorbitol
T	Tapioca starch
T_A_	Activation temperature
TC	Thermochromic

## Appendix A

**Table A1:** Dunn’s post hoc test with Bonferroni correction.

**Coating Abbreviation**	**Bursting Strength**					
	**0** **R: 5.00**	**T** **R: 35.50**	**T/S** **R: 47.11**	**T/F5/A5** **R: 38.28**	**T/F5/A2** **R: 34.72**	**T/F5/A5/S** **R: 36.89**
T	0.008737					
T/S	0.000023	1.000000				
T/F5/A5	0.002469	1.000000	1.000000			
T/F5/A2	0.012231	1.000000	1.000000	1.000000		
T/F5/A5/S	0.004702	1.000000	1.000000	1.000000	1.000000	
T/F5/A2/S	0.269667	1.000000	0.358396	1.000000	1.000000	1.000000
	**Tensile Strength MD**					
	**0** **R: 13.00**	**T** **R: 21.45**	**T/S** **R: 41.40**	**T/F5/A5** **R: 53.05**	**T/F5/A2** **R: 28.90**	**T/F5/A5/S** **R: 41.95**
T	1.000000					
T/S	0.037922	0.595971				
T/F5/A5	0.000227	0.010847	1.000000			
T/F5/A2	1.000000	1.000000	1.000000	0.167303		
T/F5/A5/S	0.030835	0.510197	1.000000	1.000000	1.000000	
T/F5/A2/S	0.001799	0.056774	1.000000	1.000000	0.612834	1.000000
	**Tensile Strength CD**					
	**0** **R: 5.65**	**T** **R: 28.85**	**T/S** **R: 42.25**	**T/F5/A5** **R: 44.20**	**T/F5/A2** **R: 46.60**	**T/F5/A5/S** **R: 46.00**
T	0.226808					
T/S	0.001215	1.000000				
T/F5/A5	0.000479	1.000000	1.000000			
T/F5/A2	0.000143	1.000000	1.000000	1.000000		
T/F5/A5/S	0.000195	1.000000	1.000000	1.000000	1.000000	
T/F5/A2/S	0.026983	1.000000	1.000000	1.000000	1.000000	1.000000
	**Elongation at Break MD**					
	**0** **R: 5.70**	**T** **R: 20.25**	**T/S** **R: 46.00**	**T/F5/A5** **R: 49.00**	**T/F5/A2** **R: 26.25**	**T/F5/A5/S** **R: 51.65**
T	1.000000					
T/S	0.000200	0.097974				
T/F5/A5	0.000041	0.033257	1.000000			
T/F5/A2	0.502959	1.000000	0.630106	0.261065		
T/F5/A5/S	0.000009	0.011770	1.000000	1.000000	0.110408	
T/F5/A2/S	0.000029	0.025967	1.000000	1.000000	0.212910	1.000000
	**Elongation at Break MD**					
	**0** **R: 9.35**	**T** **R: 42.20**	**T/S** **R: 34.85**	**T/F5/A5** **R: 41.00**	**T/F5/A2** **R: 28.70**	**T/F5/A5/S** **R: 57.15**
T	0.006445					
T/S	0.106718	1.000000				
T/F5/A5	0.010627	1.000000	1.000000			
T/F5/A2	0.703439	1.000000	1.000000	1.000000		
T/F5/A5/S	0.000003	1.000000	0.299828	1.000000	0.037221	
T/F5/A2/S	0.093044	1.000000	1.000000	1.000000	1.000000	0.338458
	**Folding Endurance**					
	**0** **R: 24.25**	**T** **R: 35.50**	**T/S** **R: 40.60**	**T/F5/A5** **R: 60.80**	**T/F5/A2** **R: 25.25**	**T/F5/A5/S** **R: 18.50**
T	1.000000					
T/S	1.000000	1.000000				
T/F5/A5	0.001244	0.114214	0.555554			
T/F5/A2	1.000000	1.000000	1.000000	0.001970		
T/F5/A5/S	1.000000	1.000000	0.318628	0.000070	1.000000	
T/F5/A2/S	0.703439	1.000000	1.000000	1.000000	0.919362	0.122183
